# Identification of heterogenous treatment response trajectories to anti-IL6 receptor treatment in rheumatoid arthritis

**DOI:** 10.1038/s41598-020-70942-x

**Published:** 2020-08-18

**Authors:** J. P. M. Blair, A.-C. Bay-Jensen, M. H. Tang, P. Frederiksen, C. Bager, M. Karsdal, S. Brunak

**Affiliations:** 1 Faculty of Health and Medical Sciences, University of Copenhagen, Copenhagen, Denmark; 2 ProScion, Herlev Hovedgade 205-207, 2730 Herlev, Denmark; 3 ImmunoScience, Nordic Bioscience, Biomarkers and Research, Herlev, Denmark

**Keywords:** Rheumatoid arthritis, Machine learning

## Abstract

Rheumatoid arthritis (RA) is a chronic inflammatory disease with fluctuating course of progression. Despite substantial improvement in treatments in recent years, treatment response is still not guaranteed. The aim of this study was to identify variation in Disease Activity Score 28 (DAS28) of RA patients in response to Tocilizumab, and to investigate both molecular and clinical factors influencing response. Clinical and biochemical data for 485 RA patients receiving Tocilizumab in combination with methotrexate were extracted from the LITHE phase III clinical study (NCT00106535), and post-hoc analysis conducted. Latent class mixed models were used to identify statistically distinct trajectories of DAS28 after the initiation of treatment. Biomarker measurements were then analysed cross-sectionally and temporally, to characterise patients by serological biomarkers and clinical factors. We identified three distinct trajectories of drug response: class 1 (n = 85, 17.5%), class 2 (n = 338, 69.7%) and class 3 (n = 62, 12.8%). All groups started with high DAS28 on average (DAS28 > 5.1). Class 1 showed the least reduction in DAS28, with significantly more patients seeking escape therapy (*p* < 0.001). Class 3 showed significantly higher rates of improvement in DAS28, with 58.1% achieving ACR response levels compared to 2.4% in class 1 (*p* < 0.0001). Biomarkers of inflammation, MMP-3, CRP, C1M, showed greater reduction in class 3 compared to the other classes. Identification of more homogenous patient sub-populations of drug response may allow for more targeted therapeutic treatment regimens and a better understanding of disease aetiology.

## Introduction

Rheumatoid arthritis (RA) is a complex disease with fluctuating course of disease activity and progression^1^. Although treatments have improved in recent years, sustained response is still not guaranteed, with most patients failing to respond to conventional synthetic DMARD therapy, the first line of treatment. The underlying reasons for drug non-response or moderate response are multi-faceted and not well understood^2^. One of the aims of precision medicine in rheumatology, is to improve drug response, enabling more targeted treatment through better understanding of the aetiology^3,4^.

Currently, assessment of drug response in clinical drug-testing trials includes discrete scores defined by American College of Rheumatology (ACR) criteria, indicating 20%, 50% or 70% improvement in a selection of clinical parameters chosen to represent disease ailments^5^. European League Against Rheumatism (EULAR) response criteria can also be used, in which improvement in the disease activity score 28 (DAS28) identifies patients as good, moderate or bad responders.

Previous studies have identified heterogeneity in treatment response trajectories in RA patients demonstrating varying rates of progression of DAS28^6,7^. These studies identified distinct groups of disease progression, either after initiation of treatment or simply through observation. They also state that there were no distinguishing factors available at baseline which were able to distinguish between the groups, aside from higher disease activity.

One explanation of variation in drug response and disease progression may be linked to the underlying endotypic profile of the patients being treated^8–11^. An endotype is a specific subtype of a condition identified that explains the observable properties of a phenotype. Endotypes define subgroups or phenotypes based on specific cells or molecules levels in blood or other fluids, and are believed to be more specific, more accurate, way of defining subgroups^12^. It has previously been postulated that RA may consist of several endotypes^9,10^, with distinctive underlying pathological mechanisms. This in turn may alter not only the manifestation of the disease in its physical form, but also drug response, and rate of progression. Using synovial biomarkers, molecular and cellular phenotypes were identified, which were closely associated with clinical outcomes to therapies targeting different biological pathways^10^. Further investigation into the mechanisms which may influence treatment response and disease progression may pave the way for better tools for precision medicine and better patient care^3^.

The aim of this study was specifically to identify heterogeneity in response to Tocilizumab, an anti-IL6 receptor treatment, in RA patients with moderate to severe active disease, and to identify factors which may describe the heterogeneity using serological biomarkers as well as patient demographics. Through a data-driven approach, we investigated disease heterogeneity using statistical learning methods.

## Methods

### Patient data

**Figure 1:**
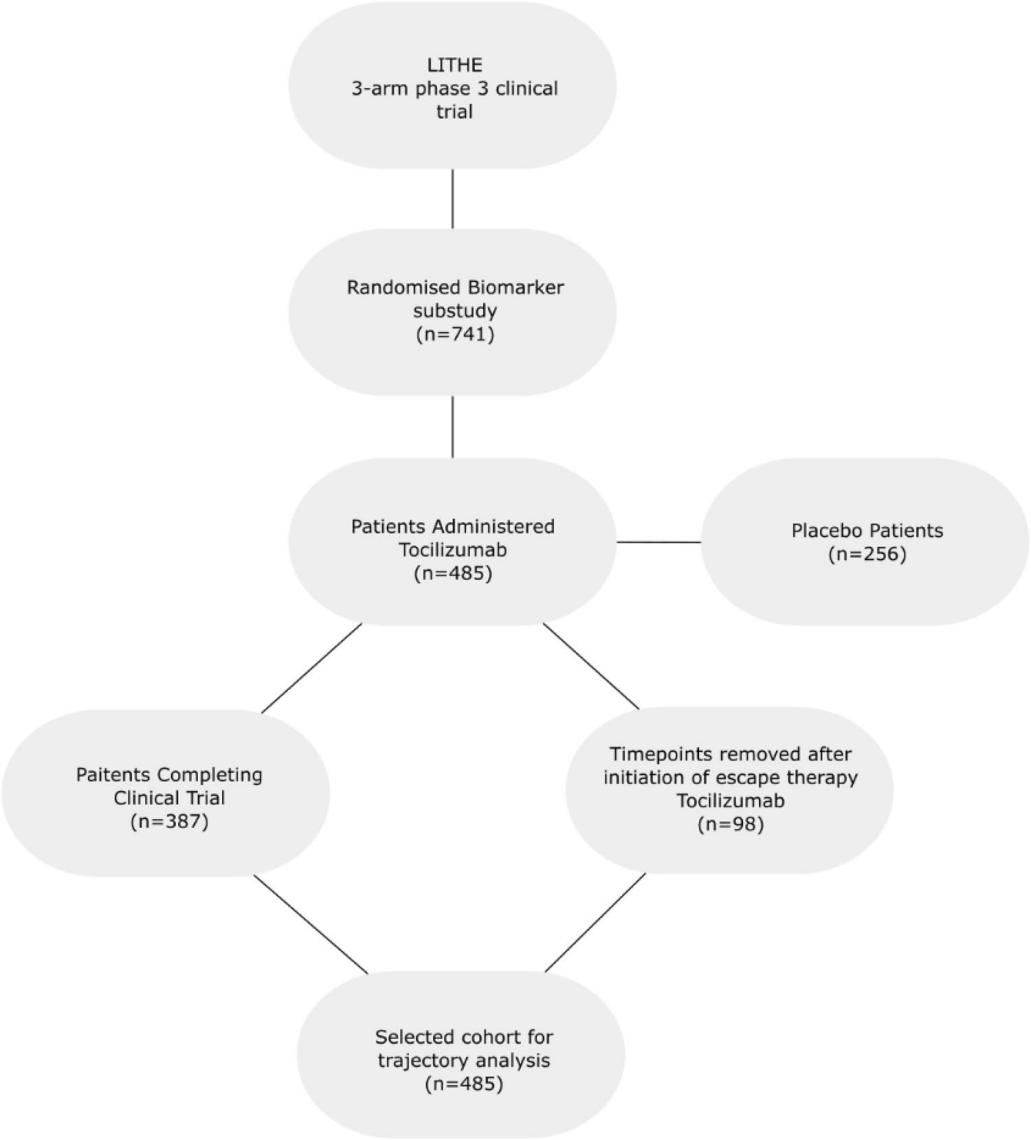
Patient selection flow diagram. Flow diagram of selection of patients and data for latent class mixture modelling to identify distinct trajectories of treatment response.

Longitudinal patient data for 485 RA patients receiving 4 or 8 mg/kg Tocilizumab in combination with MTX with a follow-up of up to 52 weeks were extracted from a biomarker sub-study^13^ of the three arm, phase III clinical trial LITHE dataset (NCT00106535)^14^. Patients receiving placebo where not included in this analysis. This cohort is comprised of patients with moderate to severe disease activity and inadequate response to DMARDs^14^. For this study, patients were selected based on a few main selection criteria which can be seen in Fig. 1. This was a post hoc and retrospective analysis of generalized data available from the clinical trials. For historical information on these clinical trials please refer to following original references^14,15^. Design and statistical plan was approved by the IRB. No new data was generated or attempted to be generated as course of the analysis. No new data was provided back to the original sponsor or PI of the project. Identifying participant information was not made available to the authors.

Demographic data (age, gender, race, disease duration, BMI) and clinical data such as previous oral steroid usage was collected at baseline (BL), upon enrolment of the study. Baseline and follow-up clinical measurements (e.g. Tender Joint Count (TJC), Swollen Joint Count (SJC), VAS pain, Health Assessment Questionnaire (HAQ), DAS28, modified Sharp Score (mTSS), etc.) were recorded every 4 weeks after baseline for 52 weeks, totalling 14 timepoints. For patients receiving escape therapy, all timepoints after initiation of escape therapy were removed (n = 98).

Endpoints were measured according to ACR and EULAR criteria, and including radiographic measurements, DAS28 disease activity scores as well as patient and physician reported outcomes.

### Latent class and statistical analysis

For this study, the lcmm package (version 1.7.9)^16^ in R was used. Different numbers of latent classes were tested ranging from 1 to 5, with the optimal number of classes chosen based on the lowest Bayesian Information Criteria and Akaike Information Criteria. Both linear and non-linear link functions were tested, with 3–5 I-splines with equidistant nodes being investigated. The number of classes chosen was also based on the percentage of the total patient cohort in each class being above 10% to avoid under-populated groups, and the posterior probability being over 0.7, ensuring a high degree of confidence in class membership. These heuristics can be seen in Table 1. Ultimately, three latent classes with 3 knots were chosen since metrics used were extremely close, and more differentiation was sought. Treatment dose and baseline oral steroid use were used as fixed effects in the model. Other variables such as age and sex were omitted due to low statistical importance. The nature of mixed effects models inherently allows for missing data meaning no imputation was performed.

**Table 1:** Parameter selection criteria for latent class modelling.

	2-Groups	3-Groups	4-Groups	5-Groups
**Linear**				
BIC	15,476.3	15,430.6	15,501.8	15,507.8
AIC	15,430.25	15,489.2	15,430.7	15,424.1
%	35/64	35.7/7.8/56.5	6.6/35.5/54.0/3.9	35.5/12.4/12.2/29.1/10.9
PP	0.79/0.84	0.74/0.6/0.77	0.69/0.74/0.76/0.72	0.80/0.90/0.61/0.73/0.87
**3 Knots**				
BIC	15,122.8	15,133.4	14,148.7	15,166.8
AIC	15,064.2	15,062.2	15,065.0	15,070.6
%	73.2/27.8	69.9/17.3/12.8	36.5/3.9/47.6/12.0	35.5/3.5/47.0/13.0/1.0
PP	0.77/0.69	0.74/0.72/0.79	0.65/0.67/0.68/0.78	0.65/0.67/0.64/0.76/0.51
**4 Knots**				
BIC	15,125.5	15,139.8	15,155.3	15,173.4
AIC	15,062.5	15,064.4	15,067.4	15,073.0
%	88.5/11.6	70.3/16.9/12.8	36.5/3.9/47.6/12.0	35.3/3.5/47.4/13.0/0.8
PP	0.88/0.72	0.74/0.72/0.79	0.64/0.67/0.68/0.78	0.64/0.67/0.64/0.76/0.50

Criteria for selection of number of groups and knots to be used in latent class analysis, in which BIC and AIC should be minimised, posterior probability of class membership (PP) should be > 0.7 for all classes, and % of total population should not be below 10%.

In each distinct trajectory group identified, baseline clinical measurements were then used in order to characterise patients by serological biomarker levels, and clinical traits. Follow-up measurements were also used to describe later timepoints of each groups response to treatment including Disease Activity Score 28. Chi-squared tests were used for non-numeric values whilst ANOVA was used for numeric measurements. In the case data was missing for a patient, statistics were computed omitting these patients, with n reported in Table 2. ACR and EULAR response rates were then compared in each group at 20%, 50% and 70% improvement levels independently using a Chi-squared test.

**Table 2:** Patient characteristics for the whole study population and identified latent classes.

	LITHE, biomarker sub-study	Class 1	Class 2	Class 3	*P* value
N	485	85	338	62	–
***Demographics***					
Age, years	53.0 ± 12.2	52.2 ± 12.8 (85)	53.4 ± 12.1 (338)	51.8 ± 11.7 (62)	0.52
**Gender**					0.63
Male, n (%)	82 (16.9)	13 (15.3)	56 (16.6)	13 (21.0)	
Female, n (%)	403 (83.1)	72 (84.7)	282 (83.4)	49 (79.0)	
BMI, cm/kg^2^	27.7 ± 6.0 (478)	27.5 ± 6.8 (84)	27.8 ± 5.7 (333)	27.4 ± 6.5 (61)	0.838
RA duration	9.8 ± 8.3 (484)	10.3 ± 8.5 (85)	9.8 ± 8.4 (337)	9.3 ± 7.2 (62)	0.748
***Treatments***					
**Baseline steroid use**					0.73
Yes, n (%)	327 (67.4)	60 (70.6)	227 (67.2)	40 (64.5)	–
No, n (%)	158 (32.6)	25 (29.4)	111 (32.8)	22 (35.5)	–
No prev. DMARD/anti TNF	1.7 ± 1.5 (485)	1.8 ± 1.5 (85)	1.7 ± 1.5 (338)	1.7 ± 1.3 (62)	0.856
**Treatment dose**					0.18
4 mg/kg (%)	240	39 (16.2)	177 (73.4)	25 (10.4)	–
8 mg/kg (%)	245	46 (18.9)	162 (66.0)	25 (15.1)	–
**Disease activity BL**					
VAS pain score	54.2 ± 22.3 (481)	57.59 ± 19.8 (84)	53.57 ± 22.3 (336)	53.34 ± 25.1 (61)	0.32
HAQ	1.5 ± 0.6 (443)	1.57 ± 0.6 (79)	1.46 ± 0.6 (308)	1.58 ± 0.6 (56)	0.19
Sharp score	28.3 ± 29.2 (466)	30.92 ± 29.7 (85)	26.89 ± 29.2 (323)	32.34 ± 27.7 (58)	0.10
DAS28	6.5 ± 0.9 (485)	6.67 ± 1 (83)	6.43 ± 0.9 (333)	6.4 ± 1 (61)	0.088
TJC	27.9 ± 14.6 (485)	31.5 ± 16.1 (85)	27.3 ± 14.3 (338)	25.8 ± 13.9 (62)	0.03
SJC	16.5 ± 9.9 (485)	18.1 ± 11.6 (85)	16 ± 9.4 (338)	16.6 ± 10.1 (62)	0.24
**Biomarkers (log10)**					
PINP (ng/mL)	48.5 ± 26.1 (444)	50.95 ± 28.8 (78)	48.23 ± 26.3 (308)	46.99 ± 21 (58)	0.99
CTXI (ng/mL)	0.4 ± 0.2 (444)	0.41 ± 0.2 (78)	0.4 ± 0.2 (308)	0.44 ± 0.2 (58)	0.62
OC (ng/mL)	21.5 ± 13.3 (444)	21.31 ± 12.2 (78)	21.32 ± 13.6 (308)	22.89 ± 13.3 (58)	0.55
ICTP (ng/mL)	5.5 ± 2.8 (425)	6.23 ± 3.9 (74)	5.4 ± 2.4 (294)	5.46 ± 2.9 (57)	0.37
MMP3 (pmol/L)	56.1 ± 65.2 (446)	54.23 ± 57.8 (78)	51.38 ± 50 (310)	83.77 ± 119.1 (58)	0.086
CRP (mg/dL)	2.07 ± 2.5 (485)	2.16 ± 2.1 (85)	1.97 ± 2.5 (338)	2.5 ± 2.6 (62)	0.039
VICM (ng/mL)	18.0 ± 62.5 (386)	14 ± 17.7 (68) (68)	18.6 ± 71.6 (267)	20.3 ± 48.8	0.061
C1M (ng/mL)	107.2 ± 68.4 (386)	107.4 ± 67.4 (68)	105.6 ± 69.7 (267)	115.29 ± 63.6 (51)	0.51
C3M (ng/mL)	42.5 ± 22.2 (386)	41.91 ± 22.4 (68)	42.39 ± 22.7 (267)	43.65 ± 19.4 (51)	0.66

Baseline characteristics of each latent class trajectory. Data presented as mean ± SD (n) or n (%) unless indicated otherwise. In the case data was missing, this was omitted from analysis.

Longitudinal biomarker data for each patient were collected and centred to baseline levels. Linear mixed effects models were used to model change in serological biomarker levels from baseline between each of the latent classes identified. Fixed effects used were time (weeks) and latent class, and patient used as random effect. A likelihood ratio test was then used for each biomarker to identify the statistical significance of including the class variable compared to time alone.

All analysis was computed using R version 3.4.1. Mixed effects models were implemented using the lme4 package^17^.

### Consent for publication

Not applicable.

## Results

A total of 485 patients were included for latent class trajectory analysis selected from a biomarker sub-study of the LITHE clinical trial of Tocilizumab. Time points were removed for patients after they escaped the trial or switched to another therapeutic agent. This resulted in 4–14 observations per patient, over a 52-week period.

**Figure 2:**
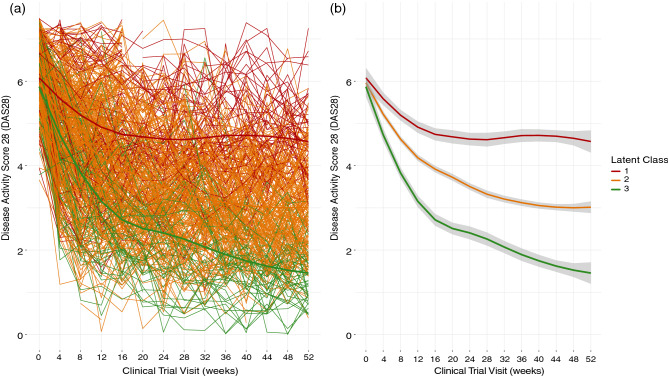
Latent trajectories of response to Tocilizumab measured by DAS28. (**A**) All patient trajectories of DAS28 in response to Tocilizumab anti-IL6 treatment, with three identified latent trajectories of DAS28 in response to Tocilizumab over a 52-week clinical trial (**B**) mean trajectory for each identified latent class from baseline to week 52.

Latent class trajectory analysis identified three distinct subgroups of patients following different trajectories of drug response. All groups started with a high DAS28, with an average 6.5 ± 0.9. On average, all three classes demonstrated a decline in disease activity, however at significantly different rates and level of change (Fig. 2) corrected for treatment dose and baseline oral steroid use.

Class 1, (17.5%, 85 patients) showed the smallest decrease in DAS28 over the 52-week period, with patients remaining above moderate disease activity status (3.2 < DAS28 < 5.1) on average after 52 weeks (DAS28 = 4.6 ± 1.1). Class 2, the largest group (69.7%, 338 patients) showed a greater decrease in the disease activity score over the course of the trial. These patients, on average reached low disease activity (2.6 < DAS28 < 3.2) but did not reach remission status after 52 weeks (DAS28 = 3.0 ± 1.1). Class 3, the smallest group (12.8%, 62 patients) showed the largest improvement in disease activity, with all patients achieving remission status after 52 weeks (DAS28 = 1.45 ± 0.6). The mean posterior probabilities of these three classes were 0.71, 0.74 and 0.80, respectively.

### Latent class characteristics

At baseline, age, gender, BMI, duration of disease, baseline steroid use and number of previous DMARDS at baseline were not significantly different (*p* > 0.05, Table 2). No differences in clinical markers of disease activity or severity, VAS Pain and radiographic Sharp score were seen at baseline either.

DAS28 showed a trend to differ at baseline with class 1, the poor responding group having higher score than the other two groups. This was likely driven by differences in CRP and TJC which were elevated in groups 3 and 1, respectively.

Whilst CRP was significantly different between groups (*p* < 0.05), other biomarkers showed little or no trend at baseline (Table 2).

The number of responders in each group according to ACR criteria was also significantly different between the three groups at week 24 and week 52, according to 20%, 50% and 70% improvement from baseline. Class 1 had significantly lower proportion of patients responding at ACR20 criteria at all time points compared to both class 2 and 3 (*p* < 0.001, Table 3). Class 3 also had significantly more patients responding at ACR70 criteria (*p* < 0.001, Table 3).

**Table 3:** Response criteria of each latent class identified.

	Lithe cohort	Class 1	Class 2	Class 3	*P* value
N	485	85	338	62	
**% ACR response**					
ACR20 (week 24)	256 (52.8)	36 (42.3)	166 (49.1)	54 (87.1)	< 0.001
ACR50 (week 24)	142 (29.3)	10 (11.7)	93 (27.5)	39 (62.9)	< 0.001
ACR70 (week 24)	64 (13.2)	3 (3.5)	36 (10.6)	25 (40.3)	< 0.001
ACR20 (week 52)	238 (49.1)	29 (34.1)	158 (46.7)	51 (82.3)	< 0.001
ACR50 (week 52)	150 (30.9)	9 (10.6)	98 (28.9)	43 (69.4)	< 0.001
ACR70 (week 52)	89 (18.3)	2 (2.4)	51 (15.1)	36 (58.1)	< 0.001
**Escape therapy**					< 0.001
Yes	99 (20.4)	38 (30.2)	59 (19.1)	2 (3.9)	
No	387 (79.6)	88 (69.8)	250 (80.9)	49 (96.1)	
EULAR response (week 24)*					< 0.001
No response	37 (10.1)	17 (16.4)	19 (10.6)	1 (1.8)	
Mod. response	170 (46.7)	57 (70.5)	105 (46.7)	8 (19.6)	
Good response	157 (43.1)	7 (13.1)	112 (42.7)	38 (78.6)	
EULAR activity (week 24)*					< 0.001
High	60 (16.3)	21 (33.3)	38 (15.3)	1 (1.8)	
Moderate	148 (40.1)	33 (52.4)	104 (41.8)	11 (19.3)	
Low	188 (19.5)	28 (11.1)	143 (21.3)	17 (21.1)	
Remission	89 (24.1)	2 (3.2)	54 (21.7)	33 (57.9)	

*Patients with missing measurements due to lack of sample or receiving escape therapy did not have a change in DAS28 calculated and were therefore omitted from this analysis.

Response criteria fulfilled by each latent class trajectory. Data presented as n (%) unless indicated otherwise. In the case data was missing, this was omitted from analysis.

### Biomarker dynamics

Serological biomarkers were chosen for the pathological mechanism or tissue they represent, central to RA; PINP, CTX-I, ICTP and OC (bone and cartilage), and MMP3, CRP, C1M and VICM (inflammation). Linear mixed effects modelling revealed biomarker change trajectories over five time points for each of the biochemical markers.

**Figure 3:**
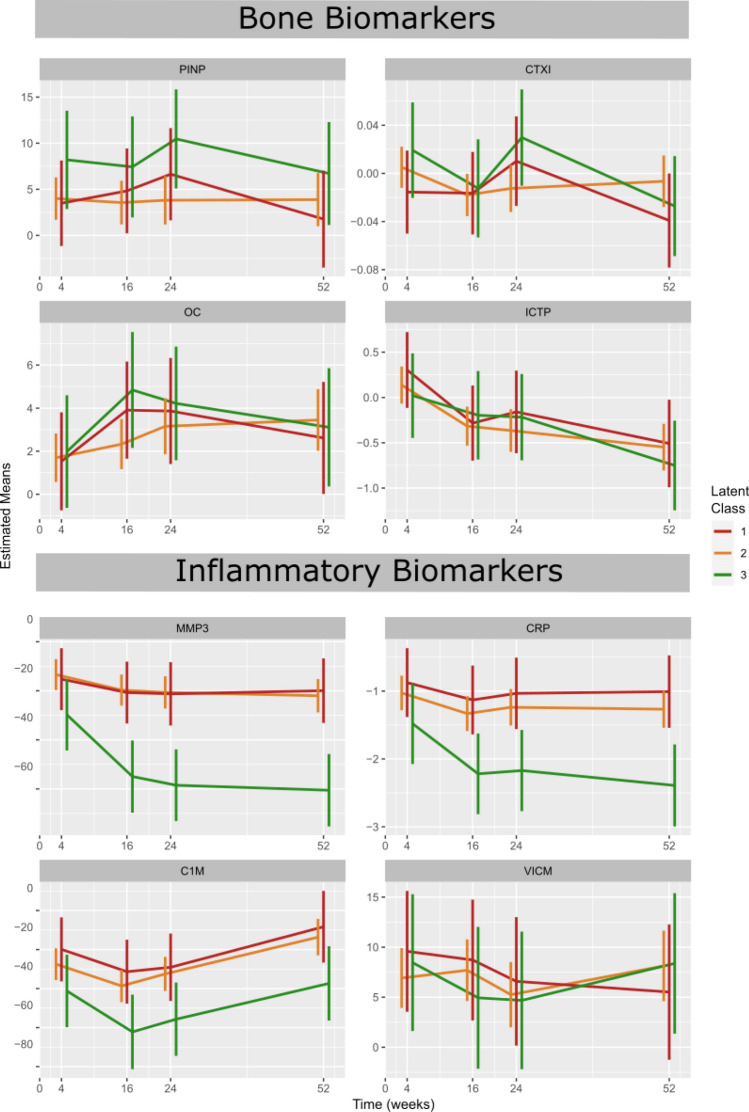
Change in biomarker levels for each latent class. Estimated means of percentage change in biomarker for PINP, CTX-I, OC and ICTP (bone), and MMP3, CRP, C1M and VICM (inflammation).

When looking at absolute change in biomarker levels from baseline, there are some differences between the three groups which could be observed. Whilst not statistically significant, markers of bone formation, PINP and OC increase more in class 3 than in class 1, which showed little sign of elevation for the first 16 weeks (Fig. 3). Patients in class 3 also demonstrate a more rapid decline in OC and ICTP than those in class 1 (Fig. 3).

Whilst markers of bone were not significantly different between classes, change in levels of MMP3 and CRP from baseline were different between classes (*p* < 0.001 and *p* = 0.03 respectively) with class 3 being much more greatly reduced (Fig. 3). VICM levels in classes 2 and 3 followed a similar path to that of C1M, whilst class 1 showed more steady decline, although all groups showed large confidence intervals.

## Discussion

The aims of this study were to identify distinct trajectories of treatment response, and to characterise these groups by clinical and longitudinal biochemical profiles. The overreaching goal of these analyses was to gain better understanding of the dynamics of response over time to highlight that different responder endotypes exist.

We identified three classes of drug response, class one, moderate responders with sustained high levels of disease activity, class 2, also moderate responders to therapy, achieving low levels of disease activity, and class 3, adequate responders, achieving remission status on average. Class three also had significantly higher proportions of patients achieving ACR drug response (20%, 50% and 70%) as well as fewer patients having to receive escape therapy. Class 2 fit closely to the median of the data set, whilst classes 1 and 3 were very much at the extremes.

As shown by other authors, response to treatment is not a linear process, and is in fact highly heterogenous^7,18^. This gives an indication that response to treatment cannot be treated unilaterally across all patients and thus must be treated as a heterogenous process, either through the identification of temporal phenotypes as we have shown here or through sub-classification of patients into disease subtypes.

These differences in the latent classes is partially explained by their clinical and serological markers. Whilst baseline demographics did not differ between the groups, DAS28 and HAQ score were elevated in class 1 and 3 compared to 2, whilst TJC was elevated in class 1 compared to 2 and 3.

We showed that the biomarker dynamics differ significantly between the three trajectories. This gives an indication that the response profiles identified are responding differently based on molecular endotype, or indeed that their molecular signature evolves over time. There was no difference in bone marker changes between the response classes (PINP, OC, CTX-I, and ICTP, although PINP seem to increase the most in class 1. Previous studies have shown that the bone resorption markers CTX-I and ICTP are suppressed by Tocilizumab, whereas neither of the bone formation markers, PINP and OC, were^13,19^. This may indicate that response measured by DAS trajectories are disassociated from drug induced changes to bone turnover however, that bone resorption itself is associated with IL-6 receptor modulation. In contrast, the tissue inflammation markers MMP3, C1M, as well as CRP, were more suppressed in class 3 as compared to class 1 or 2. The level of suppression of MMP3 and CRP for class 1 and 2 was the same, whereas there was a trend for class 2 compared to class 1 to be more suppressed in C1M. These data indicate that there is a relationship between the turnover of connective tissue collagens, measured by C1M^20^, and the DAS28 response trajectories. Previous studies have shown linear correlation between these markers and disease activity measures, as well as with drug dose response^13,21^.

Whilst there were no clear predictive biomarkers indicating membership of a given trajectory, changes in biomarker levels indicate a difference in molecular response between patients. This is true of bone biomarkers as well as inflammatory markers. Interestingly, earlier analysis of the current data set has shown that a combination of bone and connective markers was predictive of radiographic progression more so than any of the disease activity measures—again supporting the missing link between structural burden and progression and changes in DAS or other disease activity measures^22,23^. Further research into the clinical and molecular differences between patients may allow for more targeted treatment strategies. Comorbidities and multi-morbidities play a large role in defining the underlying biology of a patient, which can have profound effects on treatment response^24,25^. Adopting a more holistic approach, incorporating multiple data sources may allow for a better understanding of disease aetiology. By tailoring clinical trial design to allow for discrepancies in patient endotypes, one may expect higher response rates and fewer adverse events.

### Strengths and limitations

This study was strengthened by the availability of serological biomarkers of tissue turnover, made available at multiple time points. This allowed a more in-depth analysis of each of the trajectories identified. Unfortunately, other variables such as ACPA and RF positivity were not available which may have given more insight in to the response trajectories identified and the underlining phenotype of the patients^26,27^. Since the data used in this study was from a randomised controlled trial, many conditions may not reflect those seen in the clinic. For that reason, it may be difficult to translate any of the findings directly to clinical use.

The use of DAS28 to profile patient’s response demonstrated the heterogeneity in a clinical trial population with varying degrees of response. This score incorporates subjective measures of pain and swelling which may increase a large degree of noise into the data. By using non-subjective measures of drug response such as imaging biomarkers, molecular disease scoring systems or radiographic scores, a truer picture of physical drug response may be obtained.

## Conclusion

Statistical learning allowed the identification of distinct trajectories of patient response to anti-IL6 treatments. Through the identification of more homogenous populations of drug response, more targeted therapeutic treatment regimens may be achieved. Through the incorporation of clinical and biochemical disease parameters, as well as initial disease severity, it maybe possible to improve on current response rates.

## Data Availability

The datasets generated and/or analysed during the current study are not publicly available due to legal and ethical reasons but are available from the corresponding author on reasonable request.

## Abbreviations

RA: Rheumatoid arthritis
DAS28: Disease activity score
MTX: Methotrexate
CIM, C3M: Matrix metalloproteinase derived fragments of type I and type III collagen
ICTP: Pyridinoline cross-linked carboxy-terminal telopeptide of type I collagen
PINP: Propeptide of type I procollagen
CRP: C-reactive protein
CTX-I: C-terminal telopeptide of type I collagen
MMP3: Matrix metalloproteinase 3
OC: Osteocalcin
TJC: Tender joint count
SJC: Swollen joint count
HAQ: Heath assessment questionnaire
